# Regulation of Meiotic Prophase One in Mammalian Oocytes

**DOI:** 10.3389/fcell.2021.667306

**Published:** 2021-05-20

**Authors:** Xiaoyi Wang, Melissa E. Pepling

**Affiliations:** Department of Biology, Syracuse University, Syracuse, NY, United States

**Keywords:** meiosis, diplotene arrest, oocyte development, synaptonemal complex, recombination, primordial follicle formation

## Abstract

In female mammals, meiotic prophase one begins during fetal development. Oocytes transition through the prophase one substages consisting of leptotene, zygotene, and pachytene, and are finally arrested at the diplotene substage, for months in mice and years in humans. After puberty, luteinizing hormone induces ovulation and meiotic resumption in a cohort of oocytes, driving the progression from meiotic prophase one to metaphase two. If fertilization occurs, the oocyte completes meiosis two followed by fusion with the sperm nucleus and preparation for zygotic divisions; otherwise, it is passed into the uterus and degenerates. Specifically in the mouse, oocytes enter meiosis at 13.5 days post coitum. As meiotic prophase one proceeds, chromosomes find their homologous partner, synapse, exchange genetic material between homologs and then begin to separate, remaining connected at recombination sites. At postnatal day 5, most of the oocytes have reached the late diplotene (or dictyate) substage of prophase one where they remain arrested until ovulation. This review focuses on events and mechanisms controlling the progression through meiotic prophase one, which include recombination, synapsis and control by signaling pathways. These events are prerequisites for proper chromosome segregation in meiotic divisions; and if they go awry, chromosomes mis-segregate resulting in aneuploidy. Therefore, elucidating the mechanisms regulating meiotic progression is important to provide a foundation for developing improved treatments of female infertility.

## Introduction: Mammalian Oocyte Development and Meiosis

**FIGURE 1:**
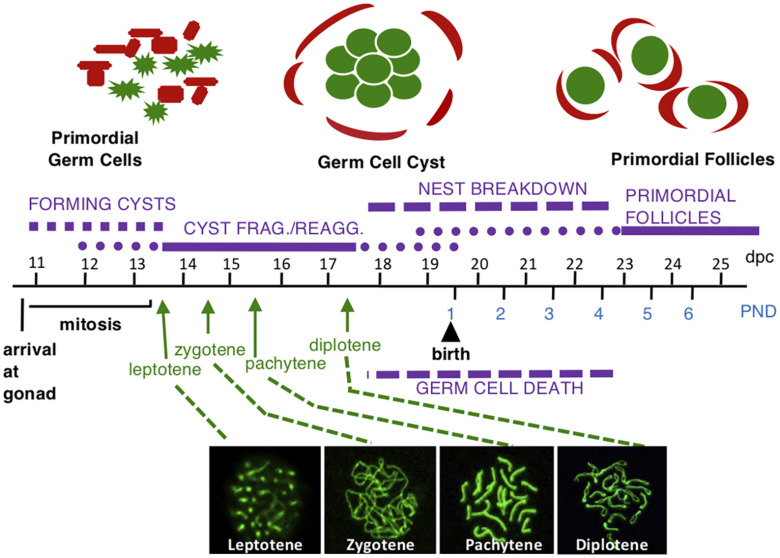
Mouse female germ cell development. Germ cells are shown in green and somatic cells are shown in red above timeline. At 10.5 dpc, germ cells migrate to the gonad and begin rapidly dividing by mitosis, forming germ cell cysts. Some cysts fragment into smaller cysts and reassociate with unrelated cysts to form nests where some cells are still connected by intercellular bridges with others associated by aggregation. Beginning at 13.5 dpc, oogonia enter meiotic prophase one to become oocytes and progress through meiotic prophase one substages: leptotene, zygotene, pachytene, and diplotene (with representative surface spread nuclei labeled with SYCP3 in green shown below the timeline). The arrow for each substage indicates the first day oocytes are found in the indicated substage. Oocytes are found in each substage for several days. The oocytes arrest at the diplotene stage starting at 17.5 dpc. At this time, germ cell nests begin to breakdown and oocytes that are not lost due to apoptosis are surrounded by somatic cells forming primordial follicles.

Meiosis is a special type of cell division that generates haploid gametes important for sexual reproduction. In meiosis, cells replicate their DNA once, followed by two rounds of division: meiosis one (MI)- a reductional division, and then meiosis two (MII)-an equational division analogous to mitotic division. In the mammalian female embryo, meiotic division of the oocyte is preceded by several rounds of mitosis. Oocytes differentiate from primordial germ cells (PGCs) that migrate to the genital ridge starting at 10.5 days post coitum (dpc) in the mouse (see Figure 1; Molyneaux et al., 2001). The germs cells divide by mitosis until 13.5 dpc and are referred to as oogonia during this time. However, cytokinesis is not complete and the oogonia remain connected by intercellular bridges in structures called germ cell cysts (Pepling and Spradling, 1998). Oogonia enter meiosis in a wave from anterior to posterior and become oocytes beginning at 13.5 dpc in the mouse (Menke et al., 2003; Bullejos and Koopman, 2004). Oocytes remain associated during fetal development though cysts may fragment and reassociate as germ cell nests (Lei and Spradling, 2013). Oocytes gradually arrest near the end of prophase one with some oocytes reaching arrest as early as 17.5 dpc and most by postnatal day (PND) 5 (Cohen et al., 2006). Concurrently, the oocytes contained in germ cell nests separate and each individual oocyte is surrounded by somatic pregranulosa cells forming structures called primordial follicles (Pepling and Spradling, 2001). As oocytes separate and follicles form, a large number of oocytes are lost by programmed cell death that in part aid in individualization of surviving cells (Greenfeld et al., 2007). In addition, other potential functions of germ cell loss have been proposed including selection for the highest quality oocytes or as support for a subset of the cyst cells (Pepling et al., 1999). More recent work has provided evidence that some oocytes play a supporting role similar to nurse cells in Drosophila (Lei and Spradling, 2016). Thus, a pool of primordial follicles each containing an oocyte arrested at the end of prophase one is established and represents the population of germ cells available for the reproductive lifespan in female mammals (Pepling, 2012). Human and mouse germ cells progress through these developmental processes analogously, except that the process in mouse is accelerated likely due to their shorter lifespan and primordial follicle formation in humans is completed during fetal development (Hartshorne et al., 2009).

In sexually mature females, follicle stimulating hormone (FSH) stimulates granulosa cell proliferation and estradiol production, inducing a preovulatory surge of luteinizing hormone (LH) which triggers meiotic resumption (Mcgee and Hsueh, 2000). This drives meiotic progression from prophase one to metaphase two. The oocyte is ovulated after the LH surge and becomes arrested in metaphase two. If fertilization occurs, the MII division is completed and followed by DNA replication in preparation for the first zygotic division; otherwise, the oocyte is passed to the uterus and disintegrates. Proper meiotic progression is important as aneuploidy, an abnormal number of chromosomes per cell occurs in at least 5% of all clinically recognized pregnancies (Hassold and Hunt, 2001). It has been estimated that women over 35 suffer from a greater risk of aneuploidy, resulting in a dramatic increase of infertility, miscarriage, and birth defects (Herbert et al., 2015).

While meiosis evolved from mitosis, novel steps were acquired that include pairing and recombination between homologous chromosomes, the inhibition of sister-chromatid separation during meiosis one (MI), and the absence of DNA replication during MII (Wilkins and Holliday, 2009). Following premeiotic DNA replication, germ cells enter an extended MI prophase which is further divided into four substages called leptotene, zygotene, pachytene, and diplotene based on cytology (Borum, 1961). During the leptotene stage, the earliest stage, chromosomes have not yet condensed and appear relatively long. In the zygotene stage, homologs begin to pair by a process called synapsis and start to condense. The pachytene stage is the third and longest stage of prophase one. By the start of the pachytene stage, the paired homologous chromosomes have become fully synapsed and by the end of this stage, chromosomes appear shortest and highly condensed. Toward the end of prophase one, homologs separate from each other marking entry into the diplotene stage. Homologous chromosomes remain physically connected at chiasmata which represent regions where crossing over has occurred during recombination which is the exchange of genetic material (Bolcun-Filas and Schimenti, 2012). It is thought that oocytes arrest in the diplotene substage because this is the most stable conformation of chromosomes as oocytes may remain at this stage until ovulation occurring months later in mice and years later in humans (Hartshorne et al., 2009). The significance of prophase one events for ensuring accurate chromosome segregation is underlined by the observation that most aneuploidies result from chromosome non-disjunction during the first meiotic division (Morelli and Cohen, 2005). This review describes recent findings on meiotic prophase one progression in mammalian oocytes up to the dictyate stage, with some reference to analogous events in mouse spermatocytes and yeast. By understanding what is known in the mouse model, we may gain insights into causes of high aneuploidy rates in human females.

## Recombination: Formation and Repair of Double-Strand Breaks

### Double-Strand Break Formation

**FIGURE 2:**
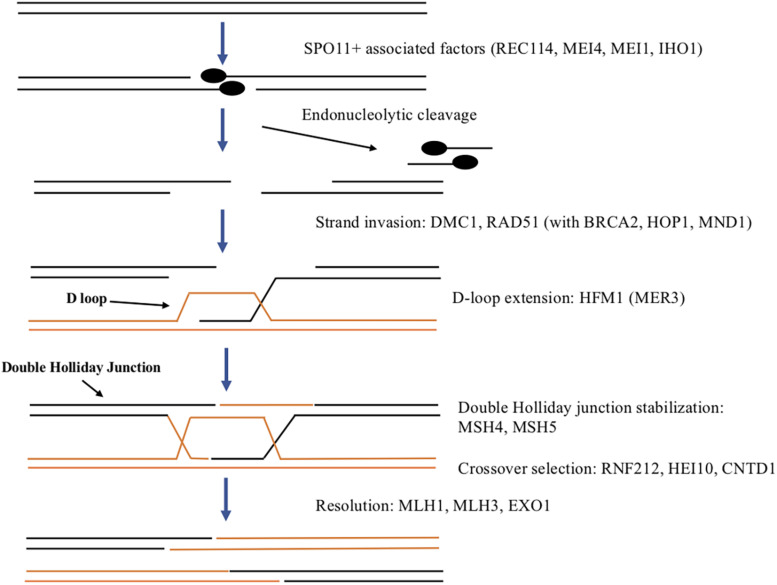
Summary of DSB formation and repair in mammals. SPO11 and associated factors including REC114, MEI4, MEI1, and IHO1 generate a double strand break to initiate recombination. Endonuclease activity results in cleavage of a small DNA fragment associated with SPO11. The resulting single-stranded DNA overhang is extended on both DNA strands by exonuclease activity. RAD51 and DMC1 along with other proteins aid in strand invasion of the homologous chromosome to begin the process of homologous recombination to repair the DSB. This forms the D-loop on the homolog that is extended by HFM1. If the other side of the DSB is “captured” a double Holliday junction is formed and stabilized by MSH4 and MSH5. This can either resolve to a crossover or a non-crossover with RNF212, HEI10, and CNTD1 involved in regulating crossover selection. Resolution to the final crossover products is promoted by MLH1, MLH3, and EXO1.

The process of recombination involves exchange of genetic material between homologous chromosomes and is initiated by generation of double-strand breaks (DSBs). In eukaryotes including mammals, DSBs are created by the SPO11 topoisomerase beginning early in prophase one (see Figure 2; Keeney, 2008). DSBs are thought to occur at recombination hotspots throughout the genome (Paigen and Petkov, 2010) and genome-wide mapping studies have identified thousands of hotspots in the mouse (Smagulova et al., 2011; Brunschwig et al., 2012). The methyltransferase, PRDM9 has been shown to be important for targeting SPO11 to recombination hotspots and is thought to direct DSB machinery to crossover sites by direct sequence-specific binding (Baudat et al., 2010; Brick et al., 2012). SPO11 creates DSBs via a transesterification reaction that cleaves the DNA backbone on both strands, with SPO11 monomers covalently attached to the 5′ ends (Keeney, 2008). SPO11-oligonucleotide (SPO11-oligo) complexes are released by endonucleolytic cleavage and serve as a by-product of DSB formation that can be used to measure DSB levels as well as distribution (Neale et al., 2005; Daniel et al., 2011). The DSBs induced by SPO11 are required for homologous chromosomes to synapse (Baudat et al., 2000; Romanienko and Camerini-Otero, 2000). In *Spo11* mutant oocytes, defects in synapsis lead to the eventual loss of all oocytes (Di Giacomo et al., 2005). Many oocytes are lost even before follicles form, though oocytes remaining undergo normal primordial follicle formation and the first wave of follicles begins to develop but within 2 months all oocytes are lost. REC114, MEI4, and MEI1 have also been implicated in DSB formation in mouse (Baudat et al., 2013). REC114 and MEI4 along with IHO1 colocalize on meiotic chromosomes and have been shown to form a complex required for DSB formation. It is thought that this complex may recruit and/or regulate the catalytic activity of SPO11 (Kumar et al., 2010, 2018; Stanzione et al., 2016) In addition, MEI1 is also required for DSB formation (Libby et al., 2003) and for MEI4 localization to meiotic chromosomes (Kumar et al., 2015).

A large number of DSBs may damage genome integrity while too few might result in deficient recombination, therefore, it is important to maintain DSB numbers within an optimal range. In yeast, orthologs of Ataxia telangiectasia mutated (ATM) and Ataxia telangiectasia and RAD3-related (ATR), members of the PI3 kinase like family of protein kinases (PIKKs) are thought to work antagonistically to regulate DSB formation during mitosis and also in meiotic prophase one (Cooper et al., 2014). Mec1, the yeast ATR ortholog is able to promote DSB formation while the yeast ATM, called Tel1 appears to negatively regulate DSBs. Similarly, in the mouse, DSB formation in meiotic prophase one significantly increases in *Atm* null spermatocytes and excessive levels of DSBs cause severe meiotic defects resulting in infertility (Lange et al., 2011). However, overexpression of ATM does not affect the number of DSBs (Modzelewski et al., 2015). In addition, ATR does not seem to affect DSB numbers in mouse spermatocytes suggesting the balance of DSBs is regulated by a different mechanism in mice (Widger et al., 2018). There is no evidence that ATM or ATR are important for regulating DSB numbers in mouse oocytes (Pacheco et al., 2019) though in both oocytes and spermatocytes, ATM and ATR play roles in the DNA damage checkpoint and elimination of germ cells (see section “Elimination of Oocytes With Defective DNA Repair or Synapsis”). Regulation of DSB formation in mammalian oocytes may involve another kinase or feedback may be provided by factors detecting synapsis.

### Double Stand Break Repair

Once DSBs are formed, they must be repaired and during this process crossovers can form. DSB repair involves end processing, strand invasion, intermediate processing and resolution with only a subset resulting in crossovers and in mouse oocytes takes about 4–5 days (Hartshorne et al., 2009). Much of our understanding of DSB repair comes from studies in yeast, flies and nematodes and the process appears to be conserved in mammals as well (reviewed in Gray and Cohen, 2016). The first step in repairing DSBs is end processing which begins with each strand of DNA being cleaved by an endonuclease releasing an oligonucleotide associated with a SPO11 monomer (see Figure 2). The cleavage is offset on one strand compared to the other leaving a two base pair overhang on each strand that is extended up to 800 bps by exonuclease activity. Recombinases RAD51 and DMC1 coat the resulting single-stranded DNA and aid in strand invasion of the homologous chromosome (Pittman et al., 1998). Several other proteins including BRCA2, HOP2, and MND1 assist RAD51 and DMC1 in strand invasion (Petukhova et al., 2005). The complementary DNA strand on the homolog is displaced forming a displacement (or D) loop. HFM1 (also called MER3) is a helicase thought to be involved in extending the D-loop (Guiraldelli et al., 2013). If a second end capture occurs, a double Holliday junction intermediate is formed and stabilized by mismatch repair proteins MSH4 and MSH5 to promote crossovers (Kneitz et al., 2000). In the mouse, several proteins participate in crossover selection including RNF212, a SUMO E3 ligase, HEI10, a ubiquitin E3 ligase, and CNTD1, a cyclin domain containing protein (Reynolds et al., 2013; Holloway et al., 2014; Qiao et al., 2014; Rao et al., 2017). Two other mismatch repair proteins MLH1 and MLH3 along with EXO1 promote resolution to crossovers (Lipkin et al., 2002). Alternatively, single end strand invasion will lead to the non-crossover pathway.

## Homolog Pairing

### DSB-Dependent Pairing

Homologous chromosome pairing starts at the zygotene stage and is essential for accurate homolog segregation during meiotic progression. It relies on DSB-dependent as well as DSB-independent pathways, and in recent years, many proteins have been identified that are involved in this regulation (see Table 1). DSBs mediate homolog pairing, and as this process culminates, homologs are coaligned ∼400 nm from each other (reviewed in Zickler and Kleckner, 2015). The coalignments consist of linkages between homologous axes which can be represented by “bridges,” each corresponding to a site of DSB-mediated inter-homolog association. Each bridge represents a nascent D-loop in which the “leading” DSB end interacts with its homologous chromosome, and therefore provides informational bias for homolog recognition, whereas the “lagging” DSB end associates with its sister chromatid. A “tentacle” hypothesis has been proposed where one end of the DSB would be released from its chromatin axis and conduct a search of the homologous chromosome (Kim et al., 2010; Panizza et al., 2011). Once the DSB has identified its partner sequence, the strands become associated and a bridge is created (Kim et al., 2010; Storlazzi et al., 2010).

**TABLE 1:** Proteins involved in homolog pairing.

Protein name	Characteristic	Functions	References
SUN1	An inner nuclear membrane protein associated with telomeres	Required for telomere-NE attachment, homologous pairing, and synapsis in spermatocytes and oocytes	Ding et al., 2007
KASH5	A dynein-dynactin binding protein locating at the outer nuclear membrane; exclusively localizes to telomeres and associates with SUN1	Essential for homologous pairing and DSB repair in spermatocytes; similar functions are assumed in oogenesis	Morimoto et al., 2012; Horn et al., 2013
TREB1	A telomere repeat-binding bouquet formation protein, meiosis-specific	Required for telomere-NE attachment and synapsis in male and female mice; homologous pairing and chromosome movement are defective in TREB1 null spermatocytes	Shibuya et al., 2014
TREB2	A telomere repeat-binding bouquet formation protein, meiosis-specific	Regulate homologous synapsis in spermatocytes and oocytes	Shibuya et al., 2015
MAJIN	Inner nuclear membrane-anchored junction protein	Essential for efficient synapsis in both male and female mice	Shibuya et al., 2015
Speedy A	A non-canonical activator of cyclin-dependent kinases; localizes to telomeres; telomere-localization domain contains distal N-terminus and Cdk2-binding Ringo domain	Mediates telomere-NE attachment, homologous pairing and synapsis in male and female mice	Tu et al., 2017

### DSB-Independent Pairing

**FIGURE 3:**
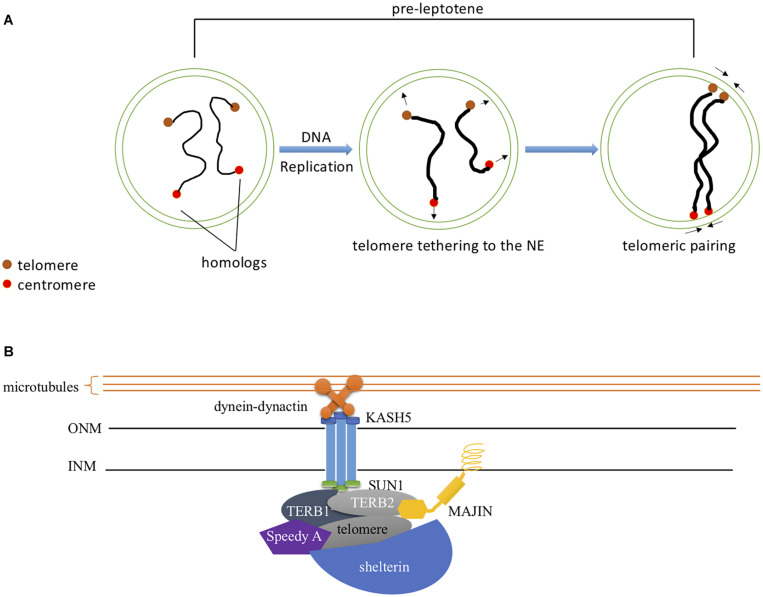
DSB-Independent Homolog Pairing **(A)** DSB-independent pairing at pre-leptotene. Telomeric homolog pairing occurs at pre-leptotene independent of DSB formation. SPO11, not through its catalytic activity, is required for this process. Interstitial pairing is promoted by telomeric pairing and its frequency decreases upon leptotene entry. **(B)** Meiotic specific protein regulation of telomere-NE attachment. Speedy A localizes to the telomere and regulates its association to the NE. TERB1/2-MAJIN attaches the telomere-shelterin complex to the nuclear envelope by anchoring MAJIN within the INM. The downstream accumulation of the SUN1-KASH5 complex to the telomere attachment site facilitates chromosome movement by linking it to the dynein-dynactin motors through KASH5.

It was widely thought that DSBs were needed for pairing of homologous chromosomes but studies from several organisms suggest that some pairing can occur before DSBs are formed (reviewed in Klutstein and Cooper, 2014). In mouse spermatocytes, a significant proportion of pairing was established before SPO11 induced DSBs (Boateng et al., 2013). In either *Mei1* mutant male mice where DSBs are absent but SPO11 expression is normal or *Spo11* mutants with defective catalytic activity, pre-leptotene pairing levels were similar to wild type. Thus, pairing also involves a DSB independent mechanism, and SPO11 catalytic activity is dispensable for this process. This involves interactions of the meiotic chromosome telomeres with the nuclear envelope (Figure 3A). The telomeres are tethered to the nuclear envelope by a protein complex called LINC (linker of nucleoskeleton and cytoskeleton) consisting of SUN1, SUN2, KASH5, and a cohesin subunit (Figure 3B; Ding et al., 2007; Schmitt et al., 2007; Adelfalk et al., 2009). KASH5 recruits dynein to the telomere attachment sites at the outer nuclear membrane and therefore mediates chromosome movements (Horn et al., 2013). The TERB1/2-MAJIN complex connects the telomeres to the LINC complex (Shibuya et al., 2015). The telomeres are capped with the Shelterin complex that protects them from damage (Palm and de Lange, 2008; Shibuya et al., 2015). TERB1/2-MAJIN binds to Shelterin thereby connecting the telomere to the nuclear envelope. An additional protein, Speedy A, has been identified as a protein required for telomere-nuclear envelope attachment in both male and female mice during meiosis (Tu et al., 2017). Mice lacking any of these meiosis-specific structural molecules are sterile (Ding et al., 2007; Morimoto et al., 2012; Horn et al., 2013; Shibuya et al., 2014, 2015; Tu et al., 2017). In mice, both telomere-nuclear envelope attachment and chromosome movements gather correct homologs together and prevent non-homologs from pairing (Koszul and Kleckner, 2009; Storlazzi et al., 2010; Tu et al., 2017). Recently, detailed interactions of pairing have been examined in mouse oocytes including how chromosomes with acrocentric telomeres interact with the nuclear envelope (Kazemi and Taketo, 2021).

Meiosis-specific cohesion proteins also regulate homologous pairing. Hopkins and colleagues identified Stromal Antigen Protein 3 (STAG3) which localizes to chromocenters (heterochromatin rich pericentrometric clusters) at the pre-leptotene stage (Hopkins et al., 2014). In *Stag3* mutant oocytes, the levels of chromosome associations within chromocenters are significantly reduced at both leptotene-like and zygotene-like stages. Homologous pairing depends on chromocenter clustering which is mediated by STAG3; therefore, STAG3 indirectly regulates inter-homolog associations in mouse oocytes.

## Synaptonemal Complex Formation and Function

### Synaptonemal Complex Assembly

**FIGURE 4:**
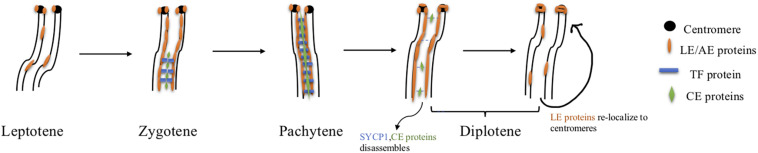
SC assembly and disassembly in mice. Axial element proteins (orange) start to load onto chromosomes at the leptotene stage. Transverse filament protein SYCP1 (blue) as well as central element proteins (green) begin to assemble at the zygotene stage. By the pachytene stage, homologous chromosomes are fully synapsed and the axial element becomes the lateral element completing the assembly of the SC. Disassembly of the SC depends on PLK1, INCENP(AURKB), and CDK1-Cyclin B1. Phosphorylated PLK1 targets central element protein TEX12, and transverse filament protein SYCP1 to promote central region disassembly. The other central element proteins are then removed from the SC. Simultaneously, both INCENP and AURKB redistribute to centromeres and facilitate lateral element protein relocation to centromere regions. CDK1 is activated by HSPA2, and active CDK1 interacts with Cyclin B1 which also targets lateral element proteins and initiates their redistribution to centromeres.

The synaptonemal complex (SC) is a proteinaceous structure that forms between homologous chromosomes and “zippers” them together in eukaryotes. In mice, SC assembly is initiated through DSB formation, which promotes homology search and synapsis (Kauppi et al., 2013). The SC is a tripartite structure composed of lateral elements (LE) on each chromosome attached to the central element (CE) by transverse filaments (TF) (see Figure 4 and Table 2). Prior to synapsis the LEs are referred to as axial elements (AE) that assemble along the chromosomes (for reviews see Fraune et al., 2012; Cahoon and Hawley, 2016). SC formation begins during the leptotene stage when SYCP2 and SYCP3 load onto the chromosome to form AEs (Yang et al., 2006). Recently, in addition to these two AE-localized proteins, five other SC proteins were identified, the TF localized protein, SYCP1 (de Vries et al., 2005) and CE proteins, SYCE1, SYCE2, SYCE3, and TEX12 (Costa et al., 2005; Hamer et al., 2006; Schramm et al., 2011). Mutations in any of these SC proteins cause a failure of synapsis in mouse spermatocytes, prophase one arrest and infertility. The situation in females is more complicated. Like the male, mutations in genes encoding CE proteins or the TF protein, SYCP1 lead to synapsis defects, meiotic arrest and infertility (de Vries et al., 2005; Bolcun-Filas et al., 2007, 2009; Hamer et al., 2008; Schramm et al., 2011). However, *Sycp2* or *Sycp3* mutant females are subfertile with smaller litter sizes. Oocytes can be fertilized and begin embryonic development but some embryos are aneuploid and do not survive (Yuan et al., 2002; Yang et al., 2006). A more recent study has identified an additional CE protein SIX6OS1, which co-localizes with SYCE1 and SYCE3 (Gomez et al., 2016). In SIX6OS1 deficient oocytes, synapsis failed and all meiocytes were arrested in a pachytene-like stage similar to the other CE mutants.

**TABLE 2:** Synaptonemal protein complex components.

Name	Time	Region	Characteristic	References
SYCP1 (synaptonemal complex 1)	Zygotene-diplotene	Transverse filament	N-terminus locates within CE and C-terminus locates within AE; recruits other CE proteins to accomplish SC assembly	Costa et al., 2005; Hamer et al., 2006; Schramm et al., 2011; Gao and Colaiácovo, 2018
SYCP2 (synaptonemal complex 2)	Leptotene-diplotene	Axial element	Interacts with C-terminus directly interacts with SYCP1; a “linker” between AE and TF	Winkel et al., 2009
SYCP3 (synaptonemal complex 3)	Leptotene-diplotene	Axial element	Major structural component of AE	Yuan et al., 2000
SYCE1 (synaptonemal complex central element 1)	Zygotene-diplotene	Central element	Recruited by SYCP1 to the CE region; interacts more directly with SYCP1	Costa et al., 2005; Hamer et al., 2006
SYCE2 (synaptonemal complex central element 2)	Zygotene-diplotene	Central element	Localization on the CE region depends on SYCP1	Costa et al., 2005
SYCE3 (synaptonemal complex central element 3)	Zygotene-diplotene	Central element	Downstream of SYCP1 but upstream of SYCE1 and -2 and enables their loading	Schramm et al., 2011
TEX12 (testis expressed sequence 12)	Zygotene-diplotene	Central element	Depends on SYCP1 to localize on the CE; co-localize with SYCE2	Hamer et al., 2006
SIX60S1	Zygotene-diplotene	Central element	Co-localizes with SYCE1 and SYCE3	Gomez et al., 2016

### SC Extension and Maintenance

Once the SC starts to assemble, it polymerizes down the length of chromosomes to fully synapse the homologs. Cells must maintain full synapsis until the completion of recombination to ensure that homologs are properly aligned and DSB repair errors are reduced (Cahoon and Hawley, 2016). In yeast, the transverse filament protein, Zip1 is important for both the extension and maintenance of the SC (Voelkel-Meiman et al., 2012, 2013; Leung et al., 2015). However, no Zip1 homolog has been identified in mammals. Recently, a protein called synaptonemal complex reinforcing element (SCRE) was found to be important for stabilizing the SC (Liu et al., 2019). In *Scre* deficient oocytes, the SC formed but synapsis could not be maintained and oocytes were lost, resulting in infertility. Therefore, SCRE maintains the integrity and stability of the SC which is essential for fertility.

### SC Disassembly

The SC disassembles during diplotene after crossovers have formed. In male mice, disassembly relies on Polo-like kinase 1 (PLK1) and Aurora B together with Inner Centromere Protein (INCENP) targeting CEs and LEs, respectively (Parra et al., 2003; Jordan et al., 2012). PLK1 phosphorylates SYCP1 and TEX12 causing subsequent central region collapse during diplotene. Following disassembly of the central region, INCENP re-localizes to centromeric heterochromatin, where Aurora B begins to localize; and results in SYCP2 and SYCP3 disassembling from LEs, both of which subsequently localize to the centromeric heterochromatin (Parra et al., 2003; Sun and Handel, 2008). In addition to PLK1 and Aurora B, CDK1- Cyclin B1 is also required for SC disassembly, as *Cdk1* deficient germ cells are arrested at the mid to late pachytene stage (Cahoon and Hawley, 2016). CDK1 is activated by interacting with the chaperone protein HSPA2, and active CDK1 further interacts with Cyclin B1 to promote LE disassembly (Zhu et al., 1997). However, the mechanism of how CDK1-Cyclin B assists in SC disassembly is not well understood. In addition, SC disassembly has not been well studied in mammalian females.

## Elimination of Oocytes With Defective DNA Repair or Synapsis

### Oocytes With Unrepaired Double Strand Breaks

During meiotic prophase one, DNA is intentionally “damaged” so that recombination can occur. Mechanisms are in place to repair this damage but if the DNA is not repaired a DNA damage response is triggered leading to the elimination of defective oocytes (for review see Gebel et al., 2020). The ATM kinase is upregulated in damaged oocytes leading to activation of CHK2 (Hirao et al., 2002). CHK2 in turn activates an oocyte-specific isoform of the p53 homolog, p63, called TAp63α (Bolcun-Filas et al., 2014; Tuppi et al., 2018). TAp63α is present in oocytes but remains inactive unless damage is detected (Kim and Suh, 2014). Recent work has shown that TAp63α upregulates proapoptotic BCL2 family members PUMA, NOXA, and BAX leading to programmed cell death of the damaged oocytes (ElInati et al., 2020). ATR, at least in spermatocytes, binds to the short stretches of single stranded DNA that appear during DSB processing (Pacheco et al., 2018; Widger et al., 2018). In females, ATR has been implicated in detecting oocytes with DSBs and activating TAp63α (Kim et al., 2019). Besides CHK2, another checkpoint kinase, CHK1 has also been found to mediate removal of damaged oocytes (Martinez-Marchal et al., 2020; Rinaldi et al., 2020).

### Oocytes With Unsynapsed Homologous Chromosomes

Mechanisms are also in place to check for and eliminate oocytes with unsynapsed homologous chromosomes (Di Giacomo et al., 2005; Cloutier et al., 2015). Oocytes lacking SPO11 cannot induce DSB formation and mice are infertile due to defective synapsis. However, the SPO11 mutants still accumulate DSBs that may be caused by activation of the LINE1 transposon (Carofiglio et al., 2013; Malki et al., 2014). The current model is that SPO11 makes DSBs and they are required for synapsis to occur. The DSBs get repaired using the homologous chromosome. HORMADs bind to unsynapsed chromosomes preventing repair using the sister chromatid and thereby promoting interaction instead with the homologous chromosome (Wojtasz et al., 2009). Interestingly, RNF212, the SUMO ligase involved in crossover control also plays a role in selecting oocytes for elimination (Qiao et al., 2018). Finally, the elimination of oocytes with unsynapsed chromosomes also depends on CHK2 and the DNA damage response pathway (Rinaldi et al., 2017). However, unlike oocytes with unrepaired DSBs, elimination of *Spo 11* mutant oocytes which contain unsynapsed chromosomes does not require BCL2 family members suggesting separate genetic mechanisms of oocyte death (ElInati et al., 2020).

## Signaling Pathways in Meiotic Prophase One

In mammalian females, oocytes are arrested in meiotic prophase one until puberty, lasting for months in mice and years in humans. Understanding the signaling events that regulate meiotic progression through prophase one is imperative to shed light on the formation of the ovarian reserve. Retinoic acid (RA) signaling initiates meiosis in mouse ovaries (Bowles et al., 2006; Koubova et al., 2006). The basis helix-loop-helix transcription factor STRA8 is activated by RA signaling (Anderson et al., 2008). This activation requires the RNA binding protein DAZL (Lin et al., 2008). RNA-seq analysis of wild-type, *Kit* mutant (which are germ cell deficient), *Dazl* mutant and *Stra8* mutant mouse fetal ovaries resulted in the identification of over 100 genes expressed during meiotic prophase one in developing female mouse ovaries (Soh et al., 2015). Almost all of these genes require DAZL for induction but only some are dependent on STRA8. Interestingly, STRA8 independent and partly independent genes encode products important for chromosome structure during meiosis such as SC proteins that would be required early in meiosis.

Steroid hormone signaling plays a role in regulating meiotic progression. Progesterone treatment of fetal mouse ovaries in organ culture resulted in a delay of progression through prophase one (Dutta et al., 2016) and this effect was mediated through the progesterone membrane receptor, PGRMC1 (Guo et al., 2016). Guo and colleagues also found that progesterone caused downregulation of cyclic adenosine monophosphate (cAMP) synthesis. An earlier study showed that inhibition of cAMP resulted in meiotic prophase one delay as well as reduced primordial follicle formation (Wang et al., 2015). In addition, they found that blocking cAMP reduced the removal and degradation of SYCP1 protein suggesting that cAMP was important for regulation of SC disassembly. Another study found that the combination of estradiol and progesterone but not progesterone alone affected prophase one progression (Burks et al., 2019). Collectively, steroid hormones have been implicated in regulating meiotic prophase one progression, and further experiments are needed to fully understand this regulation.

Phthalates are synthetic chemical esters of phthalic acid and can act as endocrine disruptors impairing reproductive function with effects on reproductive organs including the ovary (Hannon and Flaws, 2015). Neonatal exposure to the phthalate, di (2-ethylhexyl) phthalate (DEHP) reduces primordial follicle formation and increases autophagy (Mu et al., 2015; Zhang et al., 2018). The effect on primordial follicle formation was mediated through estrogen receptors which are known to be expressed in mouse ovaries at this time (Chen et al., 2009). Fetal DEHP exposure delays progression through meiotic prophase one and impairs DSB repair (Liu et al., 2017) supporting the idea that estrogens play a role in meiotic progression and can be negatively impacted by endocrine disruptors. In addition, another phthalate, dibutyl phthalate (DBP) had effects on meiotic progression and DNA repair similar to DEHP (Tu et al., 2019). Expression of DNA repair proteins including ATR was reduced and oxidative stress was induced leading to an increase in oocyte apoptosis. Another endocrine disruptor, bisphenol A (BPA) also caused meiotic prophase one defects including higher than normal recombination and synapsis failure and again these effects are thought to be through estrogen receptors (Susiarjo et al., 2007).

## Concluding Remarks

Meiotic prophase one is imperative to ensure accurate chromosome segregation as well as reproductive success. Much progress has been made in understanding the crucial checkpoints in mammalian prophase one. Interestingly, many studies have used mouse spermatocytes for elucidating meiotic events, such as DSB level regulation and homologous pairing. This is likely due to the fact that germ cells in all stages of meiotic prophase one can be obtained from adult male mice. In contrast, in females, oocytes need to be obtained during fetal stages which can be more difficult to obtain. Even though these critical events are controlled by similar genetic pathways, there are differences in checkpoint control in males and females (Morelli and Cohen, 2005). In most cases, mammalian oocytes have higher fault-tolerant rates. Therefore, while studies conducted on mouse spermatocytes contribute to our understanding of mammalian meiotic progression, there are also differences in mouse oocytes. Understanding the regulation of and progression through meiotic prophase one in oocytes and comparisons to spermatocytes will provide a more comprehensive picture of meiosis and aid in developing better female infertility treatments.

## Author Contributions

XW and MP contributed to writing and editing of this review. Both authors contributed to the article and approved the submitted version.

## Conflict of Interest

The authors declare that the research was conducted in the absence of any commercial or financial relationships that could be construed as a potential conflict of interest.
